# Transcranial direct current stimulation over the right parietal cortex improves the depressive disorder: A preliminary study

**DOI:** 10.1002/brb3.3638

**Published:** 2024-08-05

**Authors:** Xin Guo, Qilin Zhou, Yueying Lu, Zhexue Xu, Zhenye Wen, Ping Gu, Shujuan Tian, Yuping Wang

**Affiliations:** ^1^ Department of Neurology The First Hospital of Hebei Medical University Shijiazhuang Hebei China; ^2^ Department of Neurology Hebei Hospital, Xuanwu Hospital, Capital Medical University Shijiazhuang China; ^3^ Neuromedical Technology Innovation Center of Hebei Province Shijiazhuang China; ^4^ Department of Neurology Xuanwu Hospital Capital Medical University Beijing China; ^5^ Department of Neurology Fuwai Hospital, Chinese Academy of Medical Sciences Beijing China; ^6^ Department of Clinical Psychology The First Hospital of Hebei Medical University Shijiazhuang Hebei China; ^7^ Department of Rehabilitation The First Hospital of Hebei Medical University Shijiazhuang Hebei China; ^8^ Beijing Key Laboratory of Neuromodulation Beijing China; ^9^ Center for Sleep and Consciousness Disorders Beijing Institute for Brain Disorders Beijing China; ^10^ Center of Epilepsy, Beijing Institute for Brain Disorders Capital Medical University Beijing China; ^11^ Collaborative Innovation Center for Brain Disorders Capital Medical University Beijing China

**Keywords:** anxiety, depression, parietal cortex, transcranial direct current stimulation

## Abstract

**Objective:**

The right posterior parietal cortex is the core brain region of emotional processing and executive control network in the human brain, and the function of the right posterior parietal cortex is decreased in patients with major depressive disorder. This study aims to preliminarily investigate whether the excitation of the right posterior parietal cortex by transcranial direct current stimulation (tDCS) could improve their clinical symptoms.

**Methods:**

In this study, 12 patients with major depressive disorder were given tDCS treatment at Xuanwu Hospital of Capital Medical University and the First Hospital of Hebei Medical University. The stimulating electrode (anode) was placed on the patients’ right parietal cortex, whereas the reference electrode (cathode) was placed on the patients’ left mastoid. The stimulation intensity was set as 2.0 mA. The patients with depressive disorder were treated for 20 min at a time twice a day for 14 consecutive days. The severity of the clinical symptoms was evaluated using the Hamilton Depression Rating Scale‐17 (HDRS‐17) and the Hamilton Anxiety Rating Scale (HARS) at before and right after treatment.

**Results:**

The HDRS‐17 scores of patients with depressive disorder decreased significantly following the tDCS treatment compared with those before treatment (*p *< .001). Further analysis revealed that the patients’ anxiety/somatization, cognitive deficit, retardation, and sleep disorder scores all decreased significantly after the tDCS treatment (*p *< .05), although there was no significant change in their weight. Moreover, the patients’ HARS scores decreased significantly after the tDCS treatment when compared with those before treatment (*p *< .01).

**Conclusion:**

The right parietal cortex may be another key stimulation targets to improving the efficacy of tDCS treatment to the patients with major depressive disorder.

## Graphical abstract

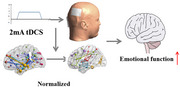

## 1 INTRODUCTION

Depressive disorder is among the most common neuropsychiatric disorders. Indeed, in China, epidemiological surveys have shown the prevalence of depressive disorder to be 2.2% and 3.3% for men and women, respectively (Liu et al., 2021). Moreover, the depressive disorder has been the second most common disease worldwide that threatens human health (Gan et al., 2021). Depressive disorder can negatively affect a person's personality, work, study, sleep, eating habits, and more. In severe cases, the depressive disorder can even be life‐threatening, with around 2%–8% of adult patients with depressive disorder dying by suicide. In fact, more than 50% of people who die by suicide worldwide suffer from depression or other emotional disorders, which places a heavy burden on families and wider society (Davis et al., 2021).

The treatment of depressive disorder has attracted increasing attention in recent decades. In addition to conventional treatment with antidepressants, neuromodulation therapy is gradually gaining prominence due to its potential to serve as another major tool for the treatment of depression (Han et al., 2019). Among the advantages of neuromodulation therapy are its noninvasive, safe, inexpensive, and easy‐to‐operate nature. As a consequence, prior studies have investigated the use of neuromodulation therapy for the treatment of neuropsychiatric diseases, such as pain, Parkinson's disease, epilepsy, tinnitus, depressive disorder, schizophrenia, and addiction (Knotkova et al., 2021; Ryvlin et al., 2021; Zhou et al., 2021). Transcranial direct current stimulation (tDCS) is a form of noninvasive neuromodulation therapy that uses scalp electrodes to deliver continuous, low‐amplitude electric currents to specific areas of the cerebral cortex. Anodal electrodes cause an increase in the excitability of the corresponding cortex by depolarizing the membrane potential of the neurons, whereas cathodal electrodes cause a decrease in the excitability of the corresponding cortex by hyperpolarizing the membrane potential of the neurons (San‐Juan, 2021). Repeated tDCS induces neuronal remodeling due to long‐term potentiation (LTP) or inhibition, which may be regulated by *N*‐methyl‐d‐aspartate receptor‐dependent mechanisms (Nitsche et al., 2006).

With the continuous development of neuroimaging and neurophysiological techniques, there is increasing evidence that major depressive disorder is a dysfunctional brain disorder caused by abnormal functioning of the brain's emotional processing and executive control networks. The dysfunction of the left dorsolateral prefrontal cortex (DLPFC) may be one of the pathophysiological mechanisms leading to major depressive disorder (Almeida et al., 2009). Currently, brain network–oriented tDCS is used to treat patients with major depressive disorder, and the stimulation target (anodal electrode) is fixed in the DLPFC. However, studies on its efficacy have reached different conclusions (Aparicio et al., 2019; Gogler et al., 2017; Loo et al., 2018). We speculated that the DLPFC may not be the only target of tDCS in the treatment of patients with major depressive disorder, and finding other key stimulation targets may be important to improving the efficacy of tDCS in the treatment of patients with major depressive disorder (Ho et al., 2014, 2015).

Numerous studies have shown the existence of attentional bias to negative stimuli in patients with major depressive disorder, suggesting that patients with depressive disorder pay excessive attention to endogenous information and neglect exogenous information. This abnormal function of the brain network may be one of the pathophysiological mechanisms leading to major depressive disorder (Kaiser et al., 2015). The parietal cortex is the core brain region of the attention network in the human brain. The dorsal region of the parietal cortex, including the superior parietal lobule and the intraparietal sulcus, is involved in top‐down attentional orienting, whereas the ventral region of the parietal cortex, including the temporoparietal association cortex, is involved in bottom‐up attentional orienting (Hutchinson et al., 2009). Abnormalities in the function of the parietal cortex may be involved in the occurrence and development of the major depressive disorder (Fales et al., 2008). Previous studies have also revealed that tDCS stimulation of the right posterior parietal cortex enhances the attention and monitoring of exogenous information (Pergolizzi & Chua, 2015).

Therefore, this study preliminarily proposed the hypothesis that the right posterior parietal cortex is one of the core brain region of emotional processing and executive control network in the human brain, and in patients with major depressive disorder, the function of the right posterior parietal cortex is reduced, leading the patients to focus too much on endogenous information and ignore exogenous information, resulting in a negative attentional bias. The excitation of the right posterior parietal cortex by the tDCS anodal electrode may correct the abnormal function of brain networks in patients with major depressive disorder, thereby improving their clinical symptoms.

## 2 METHODS

### 2.1 Subjects

Twelve patients diagnosed with depressive disorder who met the inclusion criteria and did not meet the exclusion criteria were randomly selected from the patients attending the neuropsychological clinics of Xuanwu Hospital of Capital Medical University and the First Hospital of Hebei Medical University. All interventions were authorized by the Ethics Committee of Xuanwu Hospital of Capital Medical University and the First Hospital of Hebei Medical University (approval number: 20220570). All participants provided written informed consent.

The inclusion criteria were as follows: (i) diagnosed as meeting the major depressive disorder–depressive episode diagnostic criteria according to the Diagnostic and Statistical Manual of Mental Disorders, First Edition (DSM‐V) criteria confirmed by at least two physicians at the attending level or above at the time of enrollment; (ii) aged 18–65 years old, gender not limited; (iii) had an audiovisual level sufficient to complete the examinations required for the study; (iv) did not receive electroconvulsive therapy or transcranial magnetic stimulation in the month prior to enrollment; (v) did not receive medication in the month prior to enrollment or took antidepressants stably but with poor treatment effect; (vi) Hamilton Depression Rating Scale‐17 (HDRS‐17) score ≥17 and HDRS‐17 Item 1 score ≥2; and (vii) had no contraindication to magnetic resonance imaging scans.

Moreover, the exclusion criteria were as follows: (i) had substance abuse or dependence within the 6 months prior to enrollment; (ii) had other psychiatric disorders within the 6 months prior to enrollment; (iii) had severe or unstable organic diseases; (iv) pregnant or lactating women; (v) HDRS‐17 Item 3 (suicide) score ≥3; (vi) had participated in any other clinical trial within the month prior to enrollment; and (vii) deemed by the investigator to have circumstances unsuitable for inclusion in the study.

### 2.2 Study methods

#### 2.2.1 tDCS treatment

**FIGURE 1:**
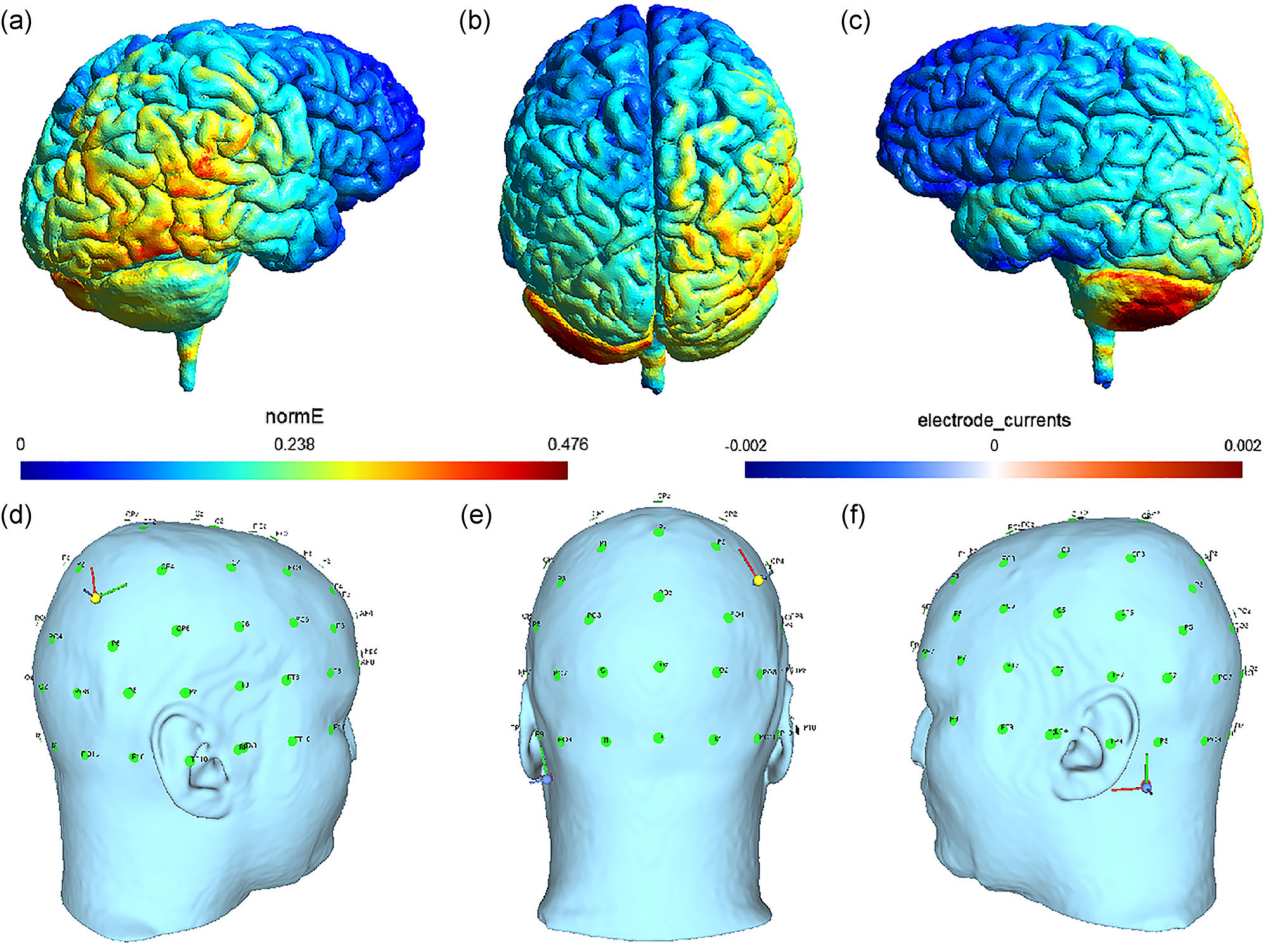
The illustration of electrode montage and estimated electric field for transcranial direct current stimulation (tDCS): (a–c) the estimated electric field in the brain from the lateral view and posterior view. The current is mainly distributed in the right superior parietal lobule, inferior parietal lobule, posterior temporal lobe, occipital lobe, left cerebellum, and occipital lobe. (d–f) electrode placement from the lateral view and posterior view. The stimulating electrode (anode) was placed on the patients' right parietal cortex (P4), whereas the reference electrode (cathode) was placed on the patients' left mastoid.

An NS‐2 multichannel electrical nerve stimulator and a low‐polarization and low‐impedance electrode patch (Beijing Yunshen Technology Co., Ltd.) were used in this study. More specifically, the electrode sheet was a spongelike electrode with a size of 7 cm × 5 cm = 35 cm^2^. The stimulating electrode (anode) was placed on the patients’ right parietal cortex (P4), whereas the reference electrode (cathode) was placed on the patients’ left mastoid. The stimulation intensity was set as 2.0 mA. The patients with depressive disorder were treated for 20 min at a time twice a day for 14 consecutive days. Patients received 28 sessions in total per course of treatment. The treatment instrument was adjusted before each treatment in order to maintain the stability of parameters, and an engineer was invited in periodically to overhaul the instrument and ensure normal operation. The electric field distribution in the brain (Figure 1) was estimated using SimNIBS 3.2.6 software (Thielscher et al., 2015).

#### 2.2.2 Observation indicators

The severity of the clinical symptoms was evaluated using the HDRS‐17 and the Hamilton Anxiety Rating Scale (HARS) before and right after treatment. After treatment, the HDRS‐17 score decreases by more than 50% compared with before treatment, which means that the treatment is effective. If the score of HDRS‐17 drops to less than 8 points after treatment, it is defined as clinical remission. The collection of basic patient data and the study medical records were completed. All the scales used in patients with depressive disorder were assessed by professional physicians employed in the neuropsychology group of the Departments of Neurology at the two hospitals to ensure their objectivity and authenticity.

#### 2.2.3 Statistical analysis

Statistical Package for the Social Sciences (SPSS) version 22.0 software was used to statistically analyze all the patient data gathered in this study. The baseline data of the patients with depressive disorder were analyzed descriptively. The normality test and homogeneity of variance test were performed on all the patients’ data. Normally distributed quantitative data were represented using mean ± standard deviation, and Skewed distributed quantitative data were represented using median and interquartile range. In addition, the primary evaluation indexes were analyzed, which involved a comparison of the HDRS‐17 and HARS scores of the patients with depressive disorder before and after the tDCS treatment using the paired samples *t*‐test or Mann–Whitney *U* test. All the statistical data were tested using the two‐sided test (test level *α* = .05).

## 3 RESULTS

### 3.1 Demographic information of the patients with depressive disorder

We recruited 12 patients with depressive disorder in this study, including 7 females. Detailed information is summarized in Table 1.

**TABLE 1:** Demographic information of the patients with depressive disorder.

Variables	*N*	Gender (male/female)	Age (years)	Level of education (college and above/high school and below)	Handedness (left/right)
Subjects	12	7/5	28 (17.75)	8/4	1/11

### 3.2 The severity of clinical symptoms of the patients with depressive disorder after tDCS treatment

**FIGURE 2:**
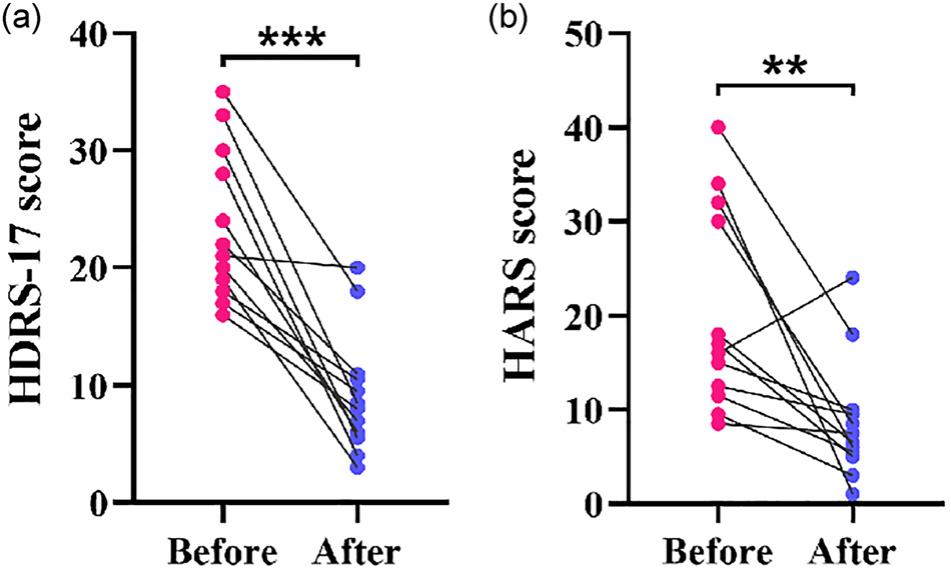
The severity of clinical symptoms of the patients with depressive disorder after transcranial direct current stimulation (tDCS) treatment: (a and b) the scores of Hamilton Depression Rating Scale‐17 (HDRS‐17) (a) and Hamilton Anxiety Rating Scale (HARS) (b) before and after tDCS treatment. **p < .01, ***p < .001.

The rate of decline in the patients’ HDRS‐17 score was found to be 59.62 ± 0.25 (%) following the tDCS treatment. There was clinical relief in seven patients, and the remission rate was 58.3%. Total effectiveness rate was 66.7%. As shown in Table 2 and Figure 2, patients with depressive disorder had higher HDRS‐17 and HARS scores and significant decline after tDCS treatment (*p* < .01).

**TABLE 2:** The severity of clinical symptoms of the patients with depressive disorder after transcranial direct current stimulation (tDCS) treatment.

Variables	Before treatment (*n* = 12)	After treatment (*n* = 12)	*t*/*Z*	*p* value
HDRS‐17 score	23.67 ± 6.30	9.00 ± 5.33	6.294	<.001
HARS score	16.50 (19.50)	6.00 (4.50)	−3.046	.002

Abbreviations: HARS, Hamilton Anxiety Rating Scale; HDRS‐17, Hamilton Depression Rating Scale‐17.

### 3.3 Values of the HDRS‐17 factors in the subjects after tDCS treatment

**FIGURE 3:**
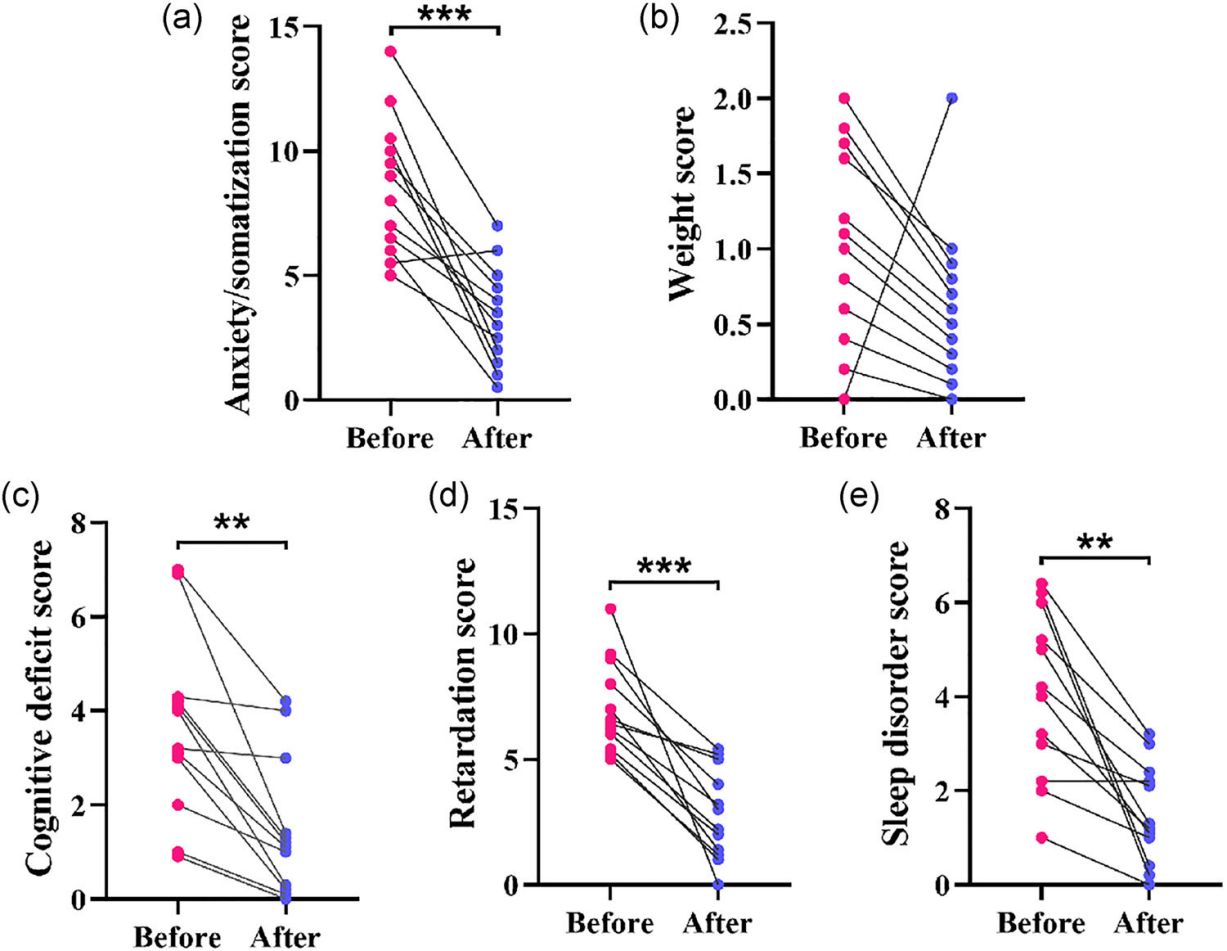
Values of the Hamilton Depression Rating Scale‐17 (HDRS‐17) factors in the patients with depressive disorder after transcranial direct current stimulation (tDCS) treatment: (a–e) the scores of anxiety/somatization (a), weight (b), cognitive deficit (c), retardation (d), and sleep disorder (e) before and after tDCS treatment. **p < .01, ***p < .001.

Compared with before treatment, significant decreases were found in the anxiety/somatization, cognitive deficit, retardation, and sleep disorder scores of the patients with depressive disorder after tDCS treatment (*p *< .01), whereas there was no significant change in their weight (*p* > .05) (Table 3 and Figure 3).

**TABLE 3:** Values of the Hamilton Depression Rating Scale‐17 (HDRS‐17) factors in the patients with depressive disorder after transcranial direct current stimulation (tDCS) treatment.

Variables	Before treatment (*n* = 12)	After treatment (*n* = 12)	*t*/*Z*	*p* value
Anxiety/Somatization score	8.58 ± 2.87	3.41 ± 2.11	5.096	<.001
Weight score	0.50 (2.00)	0.00 (0.00)	−1.733	.083
Cognitive deficit score	3.50 (1.75)	1.00 (2.50)	−2.753	.006
Retardation score	6.75 ± 2.17	2.67 ± 1.78	5.215	<.001
Sleep disorder score	3.92 ± 1.73	1.33 ± 1.07	4.640	.001

### 3.4 Occurrence of adverse events in patients with depressive disorder after tDCS treatment

All patients with depressive disorder did not have serious adverse reactions, such as nervous system complications, and did not complain of memory loss or inattention. Among them, three patients had mild skin burns, and the above symptoms were relieved after 14 days of tDCS treatment.

## 4 DISCUSSION

In this study, a total of 12 patients with depressive disorder completed tDCS therapy. Among them, the overall score of HDRS‐17 decreased to less than 8 points in seven patients after treatment, with a clinical remission rate of 58.3%. Additionally, eight patients demonstrated a reduction of more than 50% from their baseline scores, indicating a treatment effectiveness rate of 66.7%. The results of this study revealed that tDCS treatment on the right parietal lobe can lower the HAMD and HAMA scores in patients with depressive disorder. These improvements were particularly observed in factors such as anxiety/somatization, cognition, block, and sleep disturbances, whereas no effect on weight was observed. To date, there is no domestic or international literature report on the application of tDCS on the right posterior parietal cortex for treating patients with depressive disorder.

The tDCS is a noninvasive neuromodulation therapy technique with advantages in its effectiveness, tolerability, and safety. A large number of studies have demonstrated the effectiveness of tDCS in relation to the treatment of patients with depressive disorder. The tDCS is a neuromodulation therapy technique that uses scalp electrodes to deliver continuous low‐amplitude electric currents to specific cortical areas. Its exact mechanism is not yet fully understood. In fact, two possible mechanisms are currently widely accepted in the academic community. One mechanism is the immediate effect, whereby the anodal electrode of tDCS can cause an increase in the excitability of the corresponding cortex by depolarizing the resting membrane potential of the neurons, whereas the cathodal electrode can cause a decrease in the excitability of the corresponding cortex by hyperpolarizing the resting membrane potential of the neurons, thereby regulating the cortical function (Arul‐Anandam & Loo, 2009; Nitsche & Paulus, 2000). The second mechanism is the delayed effect, whereby tDCS can cause neuronal remodeling by regulating synaptic transmission, which relies on a mechanism of action similar to LTP and long‐term inhibition (Bikson et al., 2016). It has also been reported that the excitatory effects of tDCS can last from several minutes to an hour and a half (Nitsche & Paulus, 2001). Therefore, we propose that the anodal electrode of tDCS excites brain regions with abnormally weakened activity, and the cathodal electrode of tDCS inhibits brain regions with abnormally enhanced activity, regulating the abnormal function of brain networks, thus achieving the purpose of disease treatment (Hu et al., 2022; Yang et al., 2020).

Numerous studies have shown that the frontoparietal executive control network is involved in attention and emotion regulation. The parietal cortex is the core brain region of the human attention network (Hutchinson et al., 2009; Vossel et al., 2014), and its weakened activity may lead to patients with major depressive disorder paying selective attention to negative stimuli while inhibiting the processing of other stimuli (Fales et al., 2008; Olivo et al., 2018). Meanwhile, the DLPFC is involved in the advanced regulation of the subcortical emotion processing network. Compared with normal people, the activity of the left DLPFC of patients with major depressive disorder is weakened, which leads to the weakening of the top‐down endogenous inhibitory effect on the amygdala and the enhancement of the activity of the amygdala, which, in turn, enhances the bottom‐up endogenous promoting effect of the amygdala on the anterior cingulate gyrus. Thus, the activity of the DLPFC is further inhibited, resulting in negative emotional processing bias (Almeida et al., 2009). A recent study analyzing large‐scale brain network abnormalities in patients with major depressive disorder observed weakened connections within the frontoparietal network involved in the regulation of cognitive control by attention and emotion. It was also found that connections weakened between the frontoparietal network and the dorsal attention network involved in the monitoring of exogenous information. Enhanced connections within the default network involved in the perception and maintenance of endogenous information, and enhanced connections between the frontoparietal network and the default network were also reported (Kaiser et al., 2015). These results suggest that patients with depressive disorder pay excessive attention to endogenous information and neglect exogenous information. This abnormal function of the brain network may be one of the pathophysiological mechanisms leading to major depressive disorder.

Currently, brain network–oriented tDCS is used to treat patients with major depressive disorder, and the anodal electrode is fixed in the DLPFC. Most studies on DLPFC stimulation are effective (Aparicio et al., 2019; Gogler et al., 2017). However, although a substantial change of symptoms, Loo et al. (2018) reported substantial changes following placebo/sham/low dose effects had been observed previously. There are several possible reasons for the lack of greater efficacy for active tDCS in this study. First, this study may have inclusion bias, although the study methods been carefully designed to maximize blinding. Another possibility is that the stimulation on DLPFC at 2.5 mA for 30 min over 20 sessions exceeds the optimal stimulus dosage for tDCS for many participants. Our research finds that the excitation of the right posterior parietal cortex by the tDCS anodal electrode can improve clinical symptoms of patients with major depressive disorder, which may be related to correcting abnormal function of the parietal cortex. Previous studies have also revealed that tDCS stimulation of the right posterior parietal cortex enhances the attention and monitoring of exogenous information, such as the ability to recognize errors (Pergolizzi & Chua, 2015), the ability to identify hidden objects in the environment (Clark et al., 2012), and the ability to orientate spatially (Roy et al., 2015). Therefore, we speculate that the function of the right posterior parietal cortex is reduced in patients with major depressive disorder, leading the patients to focus too much on endogenous information and ignore exogenous information, resulting in a negative attentional bias, which may be one of the fundamental reasons for the occurrence and development of severe depression.

This study also found that the excitation of the right posterior parietal cortex by the tDCS anodal electrode can improve not only the depressive symptoms of patients with major depressive disorder but also their anxiety symptoms. Anxiety and depression comorbidities constitute one of the most common forms of comorbidities in clinical practice; however, their pathogenesis has not yet been elucidated. The academic community generally believes that anxiety and depression are two independent diseases that share some common pathogenic mechanisms. Previous studies have found that patients with anxiety disorders also exhibit negative attention bias, and their emotional processing networks, including the amygdala, also exhibit functional abnormalities (Etkin et al., 2009). Therefore, we hypothesized that abnormal emotional processing and executive control networks in the brain are common pathogenic mechanisms for anxiety and depression and that the right posterior parietal cortex plays a vital role in the occurrence and development of anxiety and depression comorbidities.

## 5 LIMITATIONS

This study had several limitations. First, the sample size was relatively small. Second, we should add a sham stimulation group as a control. In the subsequent studies, we urgently need to conduct large‐sample, randomized, double‐blind, controlled studies to explore the optimal stimulation parameters for achieving better treatment outcomes.

## 6 CONCLUSIONS

To sum up, the present study selected the right parietal cortex as the stimulation target for the first time and preliminarily determined that tDCS at 2.0 mA for 20 min over 28 sessions improved the depression and anxiety symptoms in patients with depressive disorder after 14 consecutive days of treatment, which may be related to the correction of the abnormal function of brain networks.

## AUTHOR CONTRIBUTIONS

**Xin Guo**: Writing—original draft; software. **Qilin Zhou**: Writing—original draft. **Yueying Lu**: Investigation. **Zhexue Xu**: Data curation; investigation. **Zhenye Wen**: Data curation. **Ping Gu**: Investigation. **Shujuan Tian**: Writing—review and editing. **Yuping Wang**: Writing—review and editing; methodology.

## CONFLICT OF INTEREST STATEMENT

The authors declare no conflicts of interest.

### PEER REVIEW

The peer review history for this article is available at https://publons.com/publon/10.1002/brb3.3638.

## Data Availability

The raw data supporting the conclusions of this article are available from the corresponding author on reasonable request.
